# Allostatic load and cardiovascular outcomes in males with prostate cancer

**DOI:** 10.1093/jncics/pkad005

**Published:** 2023-02-08

**Authors:** Nickolas Stabellini, Jennifer Cullen, Marcio S Bittencourt, Justin X Moore, Lifen Cao, Neal L Weintraub, Ryan A Harris, Xiaoling Wang, Biplab Datta, Steven S Coughlin, Jorge Garcia, John Shanahan, Nelson Hamerschlak, Kristin Waite, Nathanael R Fillmore, Martha Terris, Alberto J Montero, Jill S Barnholtz-Sloan, Avirup Guha

**Affiliations:** Graduate Education Office, Case Western Reserve University School of Medicine, Cleveland, OH, USA; Department of Hematology-Oncology, University Hospitals Seidman Cancer Center, Cleveland, OH, USA; Faculdade Israelita de Ciências da Saúde Albert Einstein, Hospital Israelita Albert Einstein, São Paulo, SP, Brazil; Department of Population and Quantitative Health Sciences, Case Western Reserve University School of Medicine, Cleveland, OH, USA; Department of Population and Quantitative Health Sciences, Case Western Reserve University School of Medicine, Cleveland, OH, USA; Case Comprehensive Cancer Center, Case Western Reserve University, Cleveland, OH, USA; Division of Cardiology, Department of Medicine, University of Pittsburgh, Pittsburgh, PA, USA; Cancer Prevention, Control, & Population Health Program, Department of Medicine, Medical College of Georgia at Augusta University, GA, USA; Department of Hematology-Oncology, University Hospitals Seidman Cancer Center, Cleveland, OH, USA; Department of Medicine, Cardiology Division, Medical College of Georgia at Augusta University, Augusta, GA, USA; Vascular Biology Center, Medical College of Georgia at Augusta University, Augusta, GA, USA; Department of Medicine, Georgia Prevention Institute, Augusta University, Augusta, GA, USA; Sport and Exercise Science Research Institute, Ulster University, Jordanstown, Northern Ireland, UK; Department of Medicine, Medical College of Georgia, Augusta University, Augusta, GA, USA; Department of Population Health Sciences, Medical College of Georgia, Augusta University, Augusta, GA, USA; Institute of Public and Preventive Health, Augusta University, Augusta, GA, USA; Department of Population Health Sciences, Medical College of Georgia, Augusta University, Augusta, GA, USA; Institute of Public and Preventive Health, Augusta University, Augusta, GA, USA; Department of Hematology-Oncology, University Hospitals Seidman Cancer Center, Cleveland, OH, USA; Cancer Informatics, Seidman Cancer Center at University Hospitals of Cleveland, Cleveland, OH, USA; Oncohematology Department, Hospital Israelita Albert Einstein, São Paulo, SP, Brazil; Trans-Divisional Research Program (TDRP), Division of Cancer Epidemiology and Genetics (DCEG), National Cancer Institute, National Institutes of Health, Bethesda, MD, USA; Cooperative Studies Program (CSP) Informatics Center, Massachusetts Veterans Epidemiology Research and Information Center (MAVERIC), VA Boston Healthcare System, Boston, MA, USA; Harvard Medical School, Boston, MA, USA; Urology Section, Department of Surgery, Veterans Affairs Medical Centers, Augusta, GA, USA; Division of Urologic Surgery, Department of Surgery, Medical College of Georgia, Augusta, GA, USA; Department of Hematology-Oncology, University Hospitals Seidman Cancer Center, Cleveland, OH, USA; Trans-Divisional Research Program (TDRP), Division of Cancer Epidemiology and Genetics (DCEG), National Cancer Institute, National Institutes of Health, Bethesda, MD, USA; Center for Biomedical Informatics and Information Technology (CBIIT), National Cancer Institute, National Institutes of Health, Bethesda, MD, USA; Department of Medicine, Case Western Reserve University School of Medicine, Cleveland, OH, USA; Cardio-Oncology Program, Ohio State University, OH, USA; Cardio-Oncology Program, Department of Medicine, Cardiology Division, Medical College of Georgia, Augusta University, Augusta, GA, USA

## Abstract

**Background:**

Cardiovascular disease (CVD) is the leading cause of death in men with prostate cancer (PC). Accumulated stress plays an important role in CVD development. The cumulative burden of chronic stress and life events can be measured using allostatic load (AL).

**Methods:**

The initial cohort included males aged 18 years and older diagnosed with PC (2005-2019). AL was modeled as an ordinal variable (0-11). Fine-Gray competing risk regressions measured the impact of precancer diagnosis AL and postdiagnosis AL in 2-year major cardiac events (MACE). The effect of AL changes over time on MACE development was calculated via piecewise Cox regression (before, and 2 months, 6 months, and 1 year after PC diagnosis).

**Results:**

We included 5261 PC patients of which 6.6% had a 2-year MACE. For every 1-point increase in AL before and within 60 days after PC diagnosis, the risk of MACE increased 25% (adjusted hazard ratio [aHR] =1.25, 95% confidence interval [CI] = 1.18 to 1.33) and 27% (aHR = 1.27, 95% CI = 1.20 to 1.35), respectively. Using AL as a time-varying exposure, the risk of MACE increased 19% (aHR = 1.19, 95% CI = 1.11 to 1.27), 22% (aHR = 1.22, 95% CI = 1.14 to 1.33), 28% (aHR = 1.28, 95% CI = 1.23 to 1.33), and 31% (aHR = 1.31, 95% CI = 1.27 to 1.35) for every 1-point increase in AL before, 2 months after, 6 months after, and 1 year after PC diagnosis, respectively.

**Conclusion:**

AL and its changes over time are associated with MACE in PC patients, suggesting a role of a biological measure of stress as a marker of CVD risk among men with PC.

Prostate cancer (PC) is the second most common type of cancer in males (1). In the United States, it represents 13.1% of all new cancer cases (2,3). Despite a high 5-year relative survival, it is one of the leading causes of death in men, having caused approximately 375 304 deaths worldwide in 2020 (1,3). Cardiovascular disease (CVD) is the leading cause of death in men with PC, excluding those that are cancer related (4-10).

There is association between cancer and CVD, through shared risk factors and biological factors (11-17). In patients with PC, androgen deprivation therapy (ADT) is associated with the development of CVD (18-21). In addition, accumulated stress also plays an important role in CVD (22,23). The cumulative burden of chronic stress and life events can be measured by allostatic load (AL), a score that computes multiple markers representing the impact of stress on cardiovascular (CV), metabolic, and immune systems and whose high values (overload) are related to poorer health outcomes and increased risk of CVD (23-26).

The role of AL and its influence on the development of CV events in patients with PC is unknown. We hypothesize that precancer diagnosis AL and its variation over time may be associated with and predict CVD events in these patients. Therefore, the primary objective of this study is to analyze the impact of AL on the development of major cardiac events (MACE) after the diagnosis of PC.

## Methods

### Data source

The study setting was the University Hospitals (UH) Seidman Cancer Center (Cleveland, Ohio, USA). Data were obtained from the UH repository, which consists of an open-source, web-based cancer data management system that integrates disparate sources of data (27). All records were deidentified, and the study was approved by the UH of Cleveland institutional review board. The information obtained was complemented with electronic health record information captured via Electronic Medical Record Search Engine (EMERSE) to obtain the most accurate and complete information per patient (28).

**Figure 1.**
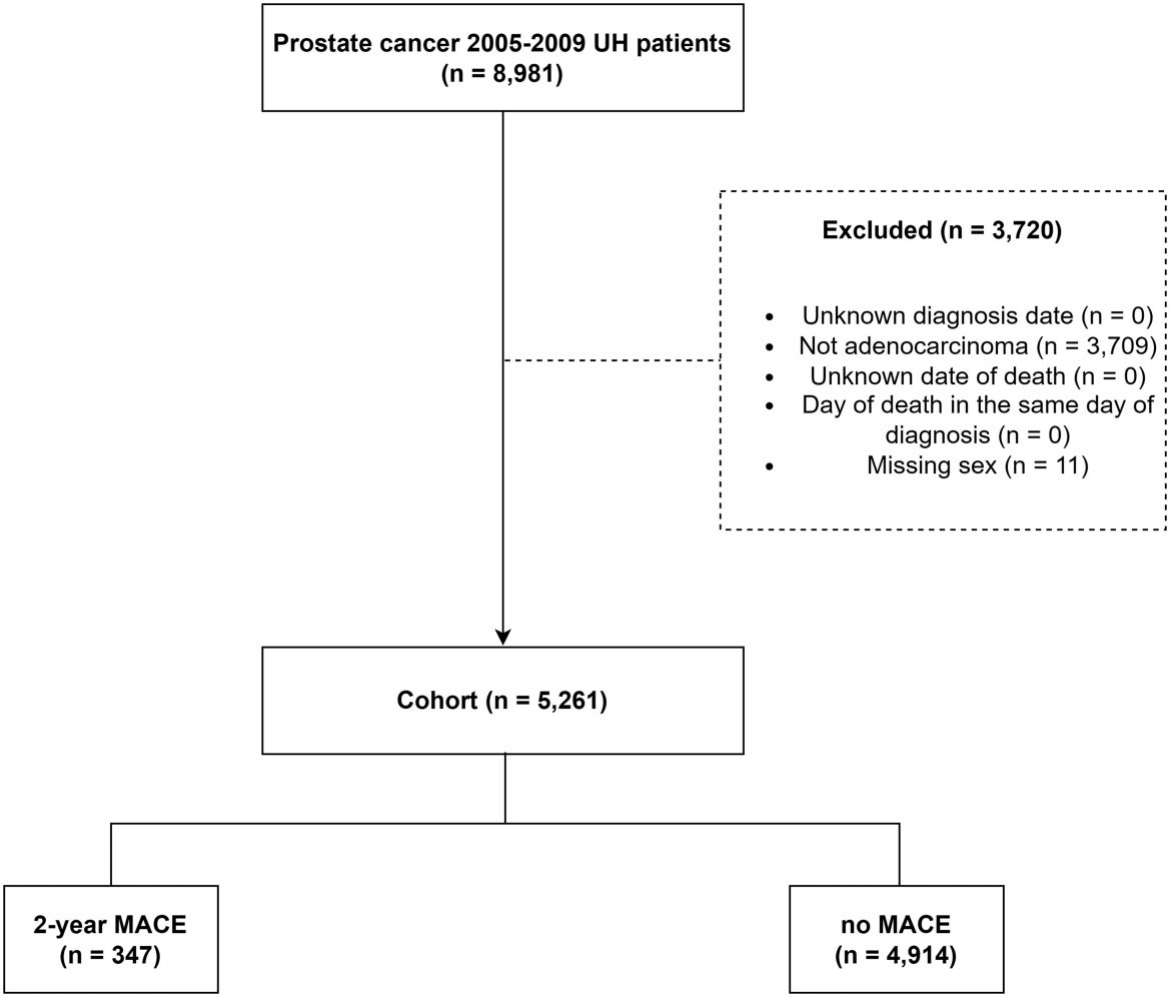
Study consort diagram detailing inclusion and exclusion criteria for prostate cancer adenocarcinoma University Hospitals (UH) population (2005-2019). The final cohort included 5261 patients of which 347 had a 2-year major cardiac event (MACE).

The cohort (see Figure 1) included males aged 18 years or older diagnosed with PC between January 1, 2005, and December 31, 2019, providing a minimum follow-up of 2 years. Patients were excluded from the analysis if they had an unknown diagnosis date and histology different than adenocarcinoma.

### Exposure

AL was used as an ordinal measure from 0 to 11 (Supplementary Figure 1, available online) using the methodology described by Rodriquez et al. (29). We assigned 1 point for the presence of each of the following: systolic blood pressure of at least 140 mmHg, diastolic blood pressure of at least 90 mmHg, heart rate of more than 100 bpm, total cholesterol of at least 240 mg/dL, high-density lipoprotein cholesterol of no more than 50 mg/dL, triglycerides of at least 150 mg/dL, glycated hemoglobin of at least 6.5, body mass index of at least 30 kg/m^2^, glucose of at least 110 mg/dL, C-reactive protein more than 3 mg/L, and interleukin-6 more than 1.8 pg/mL (26,30-34). Higher scores (overload) indicate greater physiological dysregulation.

AL was calculated as that prior to a day before cancer diagnosis, postdiagnosis (captured up to 60 days after the cancer diagnosis), 2-6 months after, 6-12 months after, and 1 year after the cancer diagnosis. There was no need for the presence of all biomarkers at all the time points for all patients. In cases with multiple measures within the time period, only those that crossed the established thresholds added 1-point to AL.

### Outcomes

The co-primary endpoints were the diagnosis and time-to-event of 2-year MACE following the cancer diagnosis. The MACE included in this study were heart failure (HF), acute coronary syndrome (ACS), atrial fibrillation (A-fib), and ischemic stroke (IS), defined using the *International Classification of Diseases* 9 and 10 (ICD-9/10) codes (Supplementary Table 1, available online) (35).

### Covariates

Demographic characteristics included age at diagnosis, self-reported race (Black, Other—any other reported race than Black or White—and White), and self-reported ethnicity (Hispanic, non-Hispanic). Risk factors were extracted based on ICD-9/10 codes (Supplementary Table 1, available online) that were presented in the patient’s electronic health record and included smoking status (yes, no, former, unknown), Elixhauser Comorbidity Index, and CVD history and risk factor (yes, no). The Elixhauser Comorbidity Index is similar to the Charlson Comorbidity Index, but the latter includes only 17 features, whereas the former includes up to 30 (36,37). The Elixhauser score has showed to be superior to the Charlson Comorbidity Score (38,39). CVD history and risk factors included hyperlipidemia, cardiomyopathy, known coronary artery disease, prior myocardial infarction, carotid disease, prior transient ischemic attack and/or stroke, and/or chronic kidney disease.

Tumor characteristics included cancer diagnosis date; biopsy Gleason score (categorized as high risk for scores ≥ 8); and tumor, nodes, and metastasis (TNM) staging group (with stage IV considered advanced stage) (40-42). Encounters included related information about hospitalizations related to MACE ICD-9/10s (length of stay in days and number of hospitalizations). Treatment characteristics included compliance to treatment (number of appointments and percent of appointments attended) and the use of a single or combination of treatments during a lifetime: radiotherapy, chemotherapy, ADTs (Supplementary Table 1, available online), and/or surgery.

### Statistical analysis

The data were presented as absolute values and percentages for categorical variables and as median and quartiles for continuous variables and stratified according to the occurrence of MACE. The Pearson χ^2^ test was used to compare categorical variables. Data distribution assumptions for continuous variables were confirmed using histograms and the Kolmogorov-Smirnov test, followed by student *t* tests for normally distributed factors and nonparametric Kruskal-Wallis tests for non-normal distributed factors. AL measures were represented via histograms (Supplementary Figure 1, available online).

**Figure 2.**
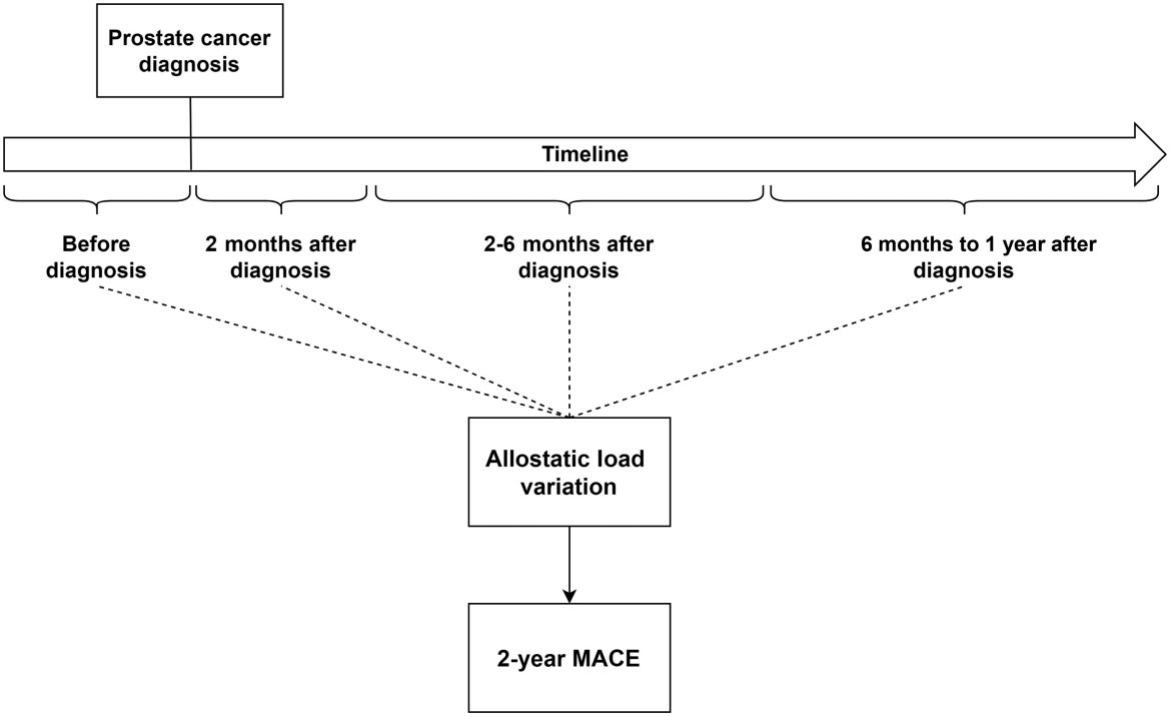
Overview of the piecewise Cox model. Four follow-up time segments related to cancer diagnosis date (before, 2 months after, 6 months after, and 1 year after) were established to account the effect of allostatic load variation in in 2-year major cardiac event (MACE).

Fine-Gray competing risk regressions were used to calculate the impact of AL pre- and postdiagnosis of cancer on 2-year MACE after confirming the model’s proportional hazards assumptions, accounting for competing risk of all-cause mortality (43). Subsequently, the effect of AL changes over time on MACE development was calculated via a piecewise Cox proportional hazards model with 4 follow-up time segments, accounting only for MACE diagnosed after AL measures (see Figure 2) (44). Subgroup analysis was performed stratifying the population by race and ethnicity (non-Hispanic Blacks, non-Hispanic Whites). Sensitivity analysis was performed stratifying the population by ADT (yes, no) because of the association of ADT with a higher risk of CVDs and by year of PC diagnosis (after 2012) to account for time-related changes in treatment and practices (18-21). Finally, the methods were replicated using AL calculated via Chen et al. (45) and Parente et al. (31) methodologies to account for variations in the calculation of AL score (26). The results were presented as hazard ratios (HRs) associated with 95% confidence intervals (CIs).

The variables selected for multivariable analysis were those that achieved a *P* value less than  .10 in univariable analyses for the primary outcome and those deemed to have clinical importance by study investigators. Correlated variables were not included simultaneously in the final models. A *P* value less than .05 was considered statistically significant, and missing values were not included in the final analysis, with a maximum reported missing rate of 4.1% in the covariates, excluding the AL biomarkers. The missing rates for each of the AL biomarkers are shown in Supplementary Table 2 (available online). All analyses were performed using RStudio software (46). The Strengthening the Reporting of Observational Studies in Epidemiology (STROBE) cohort checklist was used (47).

## Results

### Population

We included 5261 (19 840 person-years) PC patients (Table 1). The cohort’s median age was 68 (interquartile range [IQR] = 61-75) years, with a predominance of non-Hispanic (94.8%) and White (69.2%) patients. Most of the patients had an Elixhauser score between 1 and 4 (60.3%) and had no CVD history or risk factors prior to the cancer diagnosis (89.9%). Of the cases, 8% were advanced stage (IV), and 9.1% were high risk (Gleason ≥ 8). Surgery was performed in 28.9% of the cohort, and 35.7% received radiotherapy, 5.5% received chemotherapy, 1.3% received immunotherapy, and 22.4% received ADT.

**Table 1.** Prostate cancer adenocarcinoma University Hospitals (UH) population (2005-2019) description and comparison stratified by major cardiac event (MACE)^a^

Patient characteristics	Prostate cancer UH population (n = 5261)		
	MACE	No MACE	*P*
Total, No. (%)	347 (6.6)	4914 (93.4)	
Age at diagnosis, median (IQR)	74 (68-82)	67 (61-74)	<.001
Race, No. (%)			
Black	83 (23.9)	1229 (25)	.50
Other	16 (4.6)	290 (5.9)	
White	248 (71.5)	3395 (69.1)	
Ethnicity, No. (%)			
Hispanic	11 (3.2)	262 (5.3)	
Non-Hispanic	336 (96.8)	4652 (94.6)	.40
Smoking status, No. (%)			
Smoker	19 (5.5)	341 (6.9)	<.001
Never smoker	103 (29.7)	1299 (26.4)	
Former	138 (39.8)	1204 (24.5)	
Unknown	87 (25.1)	2070 (42.1)	
Elixhauser score, No. (%)			
0	3 (0.9)	173 (3.5)	<.001
1 to 4	57 (16.4)	3114 (63.4)	
≥5	287 (82.7)	1627 (33.1)	
CVD history and risk factors prediagnosis, No. (%)			
No	252 (72.6)	4479 (91.1)	<.001
Yes	95 (27.4)	435 (8.9)	
Cardiomyopathy	9 (2.6)	21 (0.4)	<.001
Coronary artery disease	37 (10.7)	114 (2.3)	<.001
Myocardial infarction	1 (0.3)	2	.48
Carotid disease	7 (2.0)	12 (0.2)	<.001
Transient ischemic attack/stroke	0	4 (0.1)	>.99
Chronic kidney disease	19 (5.5)	56 (1.1)	<.001
Hyperlipidemia	35 (10.1)	150 (3.1)	<.001
No. of CVD history and risk factors prediagnosis per patient, median (IQR)	2 (1-3)	1 (1-2)	.14
Advanced cancer stage, IV, No. (%)	21 (6.1)	401 (8.2)	.19
High risk, Gleason 8-10, No. (%)	34 (9.8)	445 (9.1)	.71
Prostate surgery, No. (%)	49 (14.1)	1471 (29.9)	<.001
Radiotherapy, No. (%)	100 (28.8)	1779 (36.2)	<.001
Chemotherapy, No. (%)	27 (7.8)	262 (5.3)	.06
ADT, No. (%)	99 (28.5)	1079 (22)	.005
No. of appointments, median (IQR)	9 (3-21)	7 (2-21)	.02
% appointments attended, median (IQR)	63.4 (33.3-80)	68.5 (50-84.6)	.007

a A total of 5261 patients were analyzed, with 347 (6.6%) in the 2-year MACE group. Other race was defined as any other self-reported race than Black or White. ADT = androgen deprivation therapy; CAD = coronary artery disease; CKD = chronic kidney disease; CVD = cardiovascular disease; IQR = interquartile range; MI = myocardial infarction.

Comparing MACE with no MACE, there was a higher median age in the MACE group (74 years, IQR = 68-82 vs 67 years, IQR = 61-74 years; *P* < .001), higher rates of ADT (28.5% vs 22%; *P* = .005), lower rates of surgery (14.1% vs 29.9%; *P* < .001) and radiotherapy (28.8% vs 36.2%; *P* < .001), and lower proportions of appointments attended (63.4%, IQR = 33.3-80% vs 68.5%, IQR = 50%-84.6%; *P* = .007).

### Outcomes

Among 5261 patients, 6.6% had a 2-year MACE, with a median time-to-event of 226 (IQR = 52-453) days after PC diagnosis. HF was diagnosed in 2.6% (median time-to-event of 256 days: IQR = 91-482 days), IS in 1.3% (median time-to-event of 249 days: IQR = 97-512 days), ACS in 2.1% (median time-to-event of 309 days: IQR = 71-489 days), and A-fib in 2.2% (median time-to-event of 222 days: IQR = 52-403 days). Each patient had a median of 1 admission because of MACE (IQR = 1-1 admissions), with a median length of stay of 2 (IQR = 1-4) days. MACEs are summarized in Supplementary Table 3 (available online).

The median AL before and post the diagnosis was 2 (IQR = 0-4), and the number rose to 3 (IQR = 1-4) after the first year. Patients diagnosed with 2-year MACE compared with those without MACE had higher median (4, IQR = 3-5 vs 1, IQR = 0-3; *P* < .001) AL before PC diagnosis, and higher median (4, IQR = 3-5 vs 3, IQR = 0-4; *P* < .001) AL after the first year. AL variation over time is represented in Supplementary Figure 1 (available online).

### Impact of AL in the development of MACE

For every 1-point increase in AL prior to PC diagnosis, we observed a 25% (adjusted HR [aHR] = 1.25, 95% CI = 1.18 to 1.33) increased risk of MACE (Table 2). One-point increase in AL post-PC diagnosis was associated with 27% (aHR = 1.27, 95% CI = 1.20 to 1.35) increased risk of MACE. Pre- and postdiagnosis AL statistically increased the risk of HF, IS, ACS, and A-fib (Table 2).

**Table 2.** Fine and Gray competing risk regressions analyzing the impact of each 1-point increase in allostatic load prior and post to the cancer diagnosis in the risk of developing a 2-year major cardiac event (MACE) and its subtypes (heart failure, ischemic stroke, acute coronary syndrome, and atrial fibrillation)^a^

Outcome	Prostate cancer population (n = 5261)			
	Precancer diagnostic allostatic load		Postdiagnosis allostatic load	
	Univariable	Multivariable	Univariable	Multivariable
	HR (95% CI)	HR (95% CI)	HR (95% CI)	HR (95% CI)
MACE	1.44 (1.39 to 1.50)	1.25 (1.18 to 1.33)	1.46 (1.40 to 1.51)	1.27 (1.20 to 1.35)
Heart failure	1.46 (1.39 to 1.54)	1.22 (1.11 to 1.34)	1.46 (1.39 to 1.54)	1.24 (1.13 to 1.36)
Ischemic stroke	1.39 (1.29 to 1.50)	1.19 (1.06 to 1.33)	1.42 (1.33 to 1.54)	1.24 (1.11 to 11.38)
Acute coronary syndrome	1.53 (1.43 to 1.63)	1.38 (1.22 to 1.55)	1.55 (1.45 to 1.65)	1.39 (1.23 to 1.57)
Atrial fibrillation	1.36 (1.27 to 1.44)	1.18 (1.07 to 1.30)	1.37 (1.29 to 1.46)	1.18 (1.08 to 1.30)

a Multivariable models were adjusted for age at diagnosis, race, smoking status, surgery, radiotherapy, androgen deprivation therapy (ADT), Elixhauser score, percent of appointments attended and cardiovascular risk factors. All tests achieved *P* < .05. CI = confidence interval; HR = hazard ratio.

Among non-Hispanic Black patients (n = 1278), 1-point increase in AL before the diagnosis increased the risk of MACE by 25% (aHR = 1.25, 95% CI = .13 to 1.38), HF by 35%, and ACS by 33% (Supplementary Table 4, available online). Among non-Hispanic White patients (n = 3478), 1-point increase in AL before the diagnosis increased the risk of MACE by 25% (aHR = 1.25, 95% CI = 1.16 to 1.35), HF by 17%, IS by 29%, ACS by 38%, and A-fib by 20% (Supplementary Table 4, available online). Similar associations were noted with postdiagnosis AL among non-Hispanic Black patients and non-Hispanic White patients (Supplementary Table 4, available online).

In patients on ADT, every 1-point increase in AL increased the risk of MACE by 26% (aHR = 1.26, 95% CI = 1.16 to 1.38), HF by 28%, IS by 20%, and ACS by 29% (Supplementary Table 5, available online). Similar associations were noted with postdiagnosis AL for patients on ADT (Supplementary Table 5, available online). For those diagnosed with PC after 2012, every 1-point increase in AL increased the risk of MACE by 10% (aHR = 1.10, 95% CI = 1.02 to 1.19; Supplementary Table 5, available online). Results from the analysis using AL calculated via Chen et al. (45) and Parente et al. (31) methodology were similar.

### Effect of AL changes in the diagnosis of MACE

Using AL as a time varying exposure, the risk of MACE increased 19% (aHR = 1.19, 95% CI = 1.11 to 1.27), 22% (aHR = 1.22, 95% CI = 1.14 to 1.33), 28% (aHR = 1.28, 95% CI = 1.23 to 1.33), and 31% (aHR = 1.31, 95% CI = 1.27 to 1.35) for every 1-point increase in AL before PC diagnosis, 2 months after, 6 months after, and 1 year after PC diagnosis, respectively (Table 3). This association persisted for HF, IS, ACS, and A-fib (Table 3).

**Table 3.** Multivariable piecewise Cox model with 4 follow-up time segments related to cancer diagnosis date (before, 0-2 months after, 2-6 months after, and 6 months to 1 year after) accounting for the effect of allostatic load (AL) variation in 2-year major cardiac event (MACE)^a^

AL and follow-up time	Prostate cancer UH population (n = 5261)				
	2-year MACE	2-year HF	2-year IS	2-year ACS	2-year A-fib
	aHR (95% CI)	aHR (95% CI)	aHR (95% CI)	aHR (95% CI)	aHR (95% CI)
AL before diagnosis	1.19 (1.11 to 1.27)	1.28 (1.24 to 1.33)	1.31 (1.26 to 1.37)	1.42 (1.37 to 1.47)	1.24 (1.20 to 1.29)
AL 2 months after diagnosis	1.22 (1.14 to 1.31)	1.29 (1.24 to 1.34)	1.32 (1.26 to 1.38)	1.42 (1.37 to 1.48)	1.25 (1.20 to 1.29)
AL 2-6 months after diagnosis	1.28 (1.23 to 1.33)	1.29 (1.24 to 1.34)	1.32 (1.26 to 1.39)	1.42 (1.37 to 1.49)	1.25 (1.20 to 1.30)
AL 6-12 months after diagnosis	1.31 (1.27 to 1.35)	1.29 (1.23 to 1.36)	1.33 (1.25 to 1.42)	1.42 (1.35 to 1.50)	1.26 (1.20 to 1.32)

a Multivariable models were adjusted for age at diagnosis, race, smoking status, surgery, radiotherapy, androgen deprivation therapy (ADT), Elixhauser score, percent of appointments attended, and cardiovascular risk factors. All tests achieved *P* < .05. ACS = acute coronary syndrome; A-fib = atrial fibrillation; aHR = adjusted hazard ratio; CI = confidence interval; HF = heart failure; IS = ischemic stroke.

In non-Hispanic Black patients, the risk of MACE increased 22% (aHR = 1.22, 95% CI = 1.06 to 1.40), 26% (aHR = 1.26, 95% CI = 1.10 to 1.45), 33% (aHR = 1.33, 95% CI = 1.22 to 1.41), and 34% (aHR = 1.34, 95% CI = 1.26 to 1.42) for every 1-point increase in AL before PC diagnosis, 2 months after, 6 months after, and 1 year after PC diagnosis, respectively, with similar associations for HF, IS, ACS, and A-fib (Supplementary Table 6, available online). In non-Hispanic White patients, the risk of MACE increased 16% (aHR = 1.16, 95% CI = 1.06 to 1.27), 20% (aHR = 1.20, 95% CI = 1.10 to 1.31), 27% (aHR = 1.27, 95% CI = 1.21 to 1.33), and 31% (aHR = 1.31, 95% CI = 1.26 to 1.36) for every 1-point increase in AL before PC diagnosis, 2 months after, 6 months after, and 1 year after PC diagnosis, respectively, with similar associations for HF, IS, ACS, and A-fib (Supplementary Table 6, available online).

Sensitivity analysis by ADT and year of diagnosis and analysis using an alternative method for AL calculation showed the similar results (Supplementary Table 6, available online).

## Discussion

This is the first study to demonstrate that a higher level of AL (chronic stress), irrespective of time before or after PC diagnosis, is associated with a 25%-30% higher risk of 2-year MACE. In addition to our main findings, the most common MACE across all subgroups was HF, and the median time-to-event was 226 days after the cancer diagnosis.

The concept of AL originated in 1993, defined as the cumulative effect of experiences in daily life that involve subtle and long-standing life situations, substantial challenges (life events), and the physiological consequences of the resulting health-damaging behaviors (eg, poor sleep, lack of exercise, smoking, alcohol consumption, and unhealthy diet) (48,49). Chemical messengers are released in response to stressors and exert cellular effects, causing systemic dysregulation of metabolic, inflammatory, and CV biomarkers, culminating in a range of health effects (48,50-54). Higher AL scores in the general population are associated with a higher risk of mortality, cognitive decline, physical function decline, and CVD, particularly coronary heart disease, ischemic heart disease, and peripheral arterial disease (22,49,55-58). Our findings demonstrate the same patterns for PC patients in which higher AL scores (pre- and post-PC diagnosis) were associated with MACE, especially HF. In addition, patients with higher AL had multiple CV risk factors such as age, higher rates of smoking, and higher Elixhauser scores, showing that AL seems to capture CV risk factors and perform well as a marker of CVD in cancer patients, much like the general population.

One of the potential differentiators of AL over current used scores (such as the Framingham risk score, Reynolds risk score) is, as it is an objective measure of chronic stress, it may have the ability to capture social determinants of health (SDOH), which the individual risk factors (eg, hypertension) are unable to capture (29,49,59). People living in adverse social economic conditions tend to experience a higher stress exposure and accumulation (60-63). In line with these descriptions, we showed lower appointment attendance rates in those with MACE, which may primarily be an effect of transportation, social support, and health-care system access for the cancer patient rather than the effect of a CV risk factor.

Racial and ethnic disparities are crucial when dealing with a measure effected by living conditions. Non-Hispanic Black men have a higher incidence of CVD and tend to live in worse conditions, experience inequalities in access to health, and accumulate a higher rate of stress, with AL already having been shown to be partially related to higher mortality in Black patients (64-67). We demonstrated, on facing disparity in the form of higher AL, non-Hispanic Black patients and non-Hispanic White patients with PC have a higher risk of MACE. Thus, biological measurement of disparities in the form of AL may help understand the role of adverse SDOH and race-related disparities differently (68).

Another sensitivity analysis considered ADT, which is the mainstay of treatment for advanced prostate tumors and is implicated in CVD (69,70). The use of ADT has been associated with increased CV risk and mortality, leading to a joint scientific statement in 2010 (20,71-73). We showed that, considering AL measures, the risk of MACE, except IS, is higher irrespective of ADT use, probably reflecting the burden caused by a cancer diagnosis and treatment. Thus, a paradigm shift in risk measurement may be warranted, where, in addition to measuring a standard set of risk measures per the 2010 statement, measures of adverse SDOH and/or AL may help in risk stratifying all patients with PC and not just those starting ADT.

On a clinical perspective, our results encourage the use of AL scores by practitioners as a marker of MACE risk in patients diagnosed with PC. Patients can have their score measured soon after diagnosis, and those characterized as high risk have a closer follow-up, with multidisciplinary participation of cardio-oncology teams, aiming at risk mitigation. Moreover, this measure as a routine in clinical practice, such as in primary care, can be a driver for the formulation of personalized support plans that reduce the impact of stressful events and, consequently, the development of risk factors and CVD. However, we emphasize that future studies should objectively analyze the superiority of the incorporation of AL over the methods and scores already used.

This study has several limitations. Our institutional database is Electronic Medical Record (EMR) based, and some of the information may be incomplete or unavailable (ie, cause of death). As a single institution, some patients may have been lost to follow-up or sought emergency care at other institutions. The time frame employed can encompass generational changes in treatment, which we hopefully mitigated with analysis of patients diagnosed after 2012. Some of the biomarkers are not routinely measured (ie, interleukin-6) and were not available at all time points for all patients. Moreover, some of these biomarkers are requested and/or measured more frequently in patients with clinical indications (eg, diabetic patients and glycated hemoglobin), which may lead to an overrepresentation of these patients in the results and may have generated lower AL levels in patients without these clinical indications. In contrast, in our analysis, we considered large time periods (before PC diagnosis, 0-2 months after PC diagnosis, 2-6 months after PC diagnosis, and 6-12 months after PC diagnosis) where only 1 read of each component was needed to account for it in AL, and the adequate availability of these markers was shown by our group (68). In the competing-risks analysis, a little number of the all-cause mortality may be comprised by deaths from cardiac events that were not diagnosed before and should be counted as MACE. However, our database integrates disparate sources, including individual detailed and longitudinal information rarely seen in other databases. In addition, the large number of patients and different statistical models with multivariable and sensitivity analysis mitigated the systematic errors pointed above. Finally, as an oncology center, we maintain a close follow-up with patients who usually come to our emergency department. Future studies should focus on multicentric designs and comparison of AL over currently used scores for CVD risk, aiming to understand the applicability of these results to other cancer types and the role of SDOH in the relationship between AL and CVD.

In conclusion, AL and its variation over time are associated with MACE, suggesting that the physiological changes and cumulative stress leading up to cancer diagnosis could serve as a marker of risk for MACE in patients with PC. Identifying reasons for higher AL, such as adverse SDOH when evaluating a patient with PC, will help identify those at the highest risk of MACE.

## Supplementary Material

pkad005_Supplementary_Data

## Data Availability

University Hospitals (UH) Seidman Cancer Center database is available at University Hospitals Cleveland Medical Center and has access restricted to researchers with Institutional Review Board (IRB) approval.
