# Supplementary material for: Allostatic load and cardiovascular outcomes in males with prostate cancer
Source: JNCI Cancer Spectr. 2023 Feb 8;7(2):pkad005. doi: 10.1093/jncics/pkad005 (PMC10005613; doi:10.1093/jncics/pkad005)
Supplement: pkad005_Supplementary_Data [file pkad005_supplementary_data.pdf]

Title: Allostatic load and cardiovascular outcomes in males with prostate cancer

Supplementary Material

Supplementary Figure 1. A. Allostatic load prior to the diagnosis. **B**. Allostatic load post-diagnosis. **C**. Allostatic load variation in the first year. **D**. Allostatic load after 1 year. Prostate cancer adenocarcinoma UH population (2005-2019). "YES" refers to patients with 2-year major cardiac event (MACE), while "NO" refers to patients without this outcome.

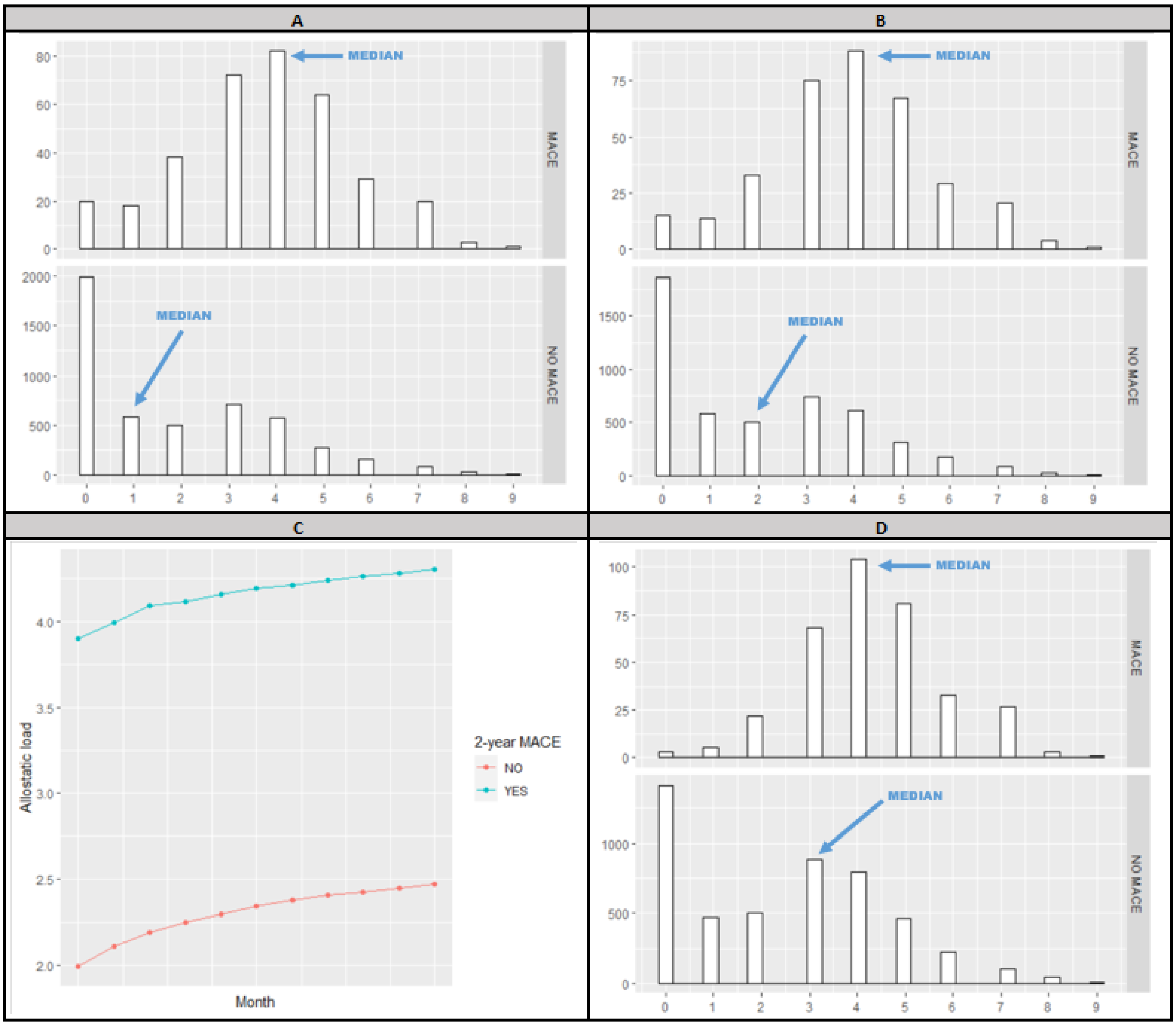

Supplementary Table 1. ICD9 and ICD10 codes used for definition of cardiac history and cardiac events diagnosis; list of medications included as categorization for ADT treatment.

Cardiovascular risk factors	ICD9	ICD10
Cardiomyopathy	425.XX	I42.XX
Known Coronary Artery Disease	414.00, 414.01, 414.02, 414.03, 414.04, 414.05, 414.06, 414.07	I25.10, I25.810, I25.811, I25.812
Prior Myocardial Infarction	412.XX	I25.2
Carotid Disease	433.10	I65.29
Prior TIA/Stroke	438.XX	I69.9XX
Chronic Kidney Disease	585.XX	N18X
Hyperlipidemia	272.X	E78X
**Events**	**ICD9**	**ICD10**
Heart Failure	428.XX, 398.9, 402.XX, 401.XX, 414.8, 425.XX,785.51	I50.XX, I09.9, I11.0, I13.0, I13.2, I25.2, I42.0, I42.5-I42.9, I43.XX, R570
Ischemic Stroke	431.XX, 435.0X, 435.1X, 435.2X, 435.3X, 435.8X, 435.9X, 433.01, 433.11, 433.21, 433.31, 433.81,	I619, G45X, I63, I66, G834, G9781

	433.91, 434.01, 434.11, 434.91, 997.01, 344.60, 344.61	
Myocardial Infarction	411.XX, 410.XX	I20.0, I21.X, I24.X
Atrial Fibrillation	427.31	I48.91
**Medication group**	**Medications included**	
ADTs	Leuprolide, Goserelin, Triptorelin, Degarelix (Firmagon), Regulolix (Orgovyx)	

Supplementary Table 2. Missing rates for each component of Allostatic Load from the dataset used in our study.

Biomarker	Missing rate
Systolic blood pressure	<1%
Diastolic blood pressure	<1%
Heart rate	<1%
Total cholesterol	<1%
High-density lipoprotein cholesterol	<1%
Triglycerides	1.2%
Glycated hemoglobin	3%
Body mass index	<1%
Glucose	<1%
C-reactive protein	11.3%
Interleukin-6	37.5%

Supplementary Table 3. Cardiac outcomes (MACE, heart failure, ischemic stroke, acute coronary syndrome, atrial fibrillation, admissions due to MACE and length of stay in days) for prostate cancer adenocarcinoma UH population (2005-2019). IQR = interquartile range.

	**Prostate Cancer UH population (n=5,261)**	
	**n (%)**	**Median time-to-event in days (IQR)**
**Major cardiac event (MACE)**	347 (6.6)	226 (52, 453)
**Heart failure (HF)**	137 (2.6)	256 (91, 482)
**Ischemic stroke (IS)**	70 (1.3)	249 (97, 512)
**Acute coronary syndrome (ACS)**	112 (2.1)	309 (71, 489)
**Atrial fibrillation (A-Fib)**	114 (2.2)	222 (52, 403)
**Admissions due to MACE per patient - median (IQR)**	1 (1, 1)	-
**Length of stay (LOS) in days per patient admitted due to CEV - median (IQR)**	2 (1, 4)	-

Supplementary Table 4. Fine and Gray competing risk regressions analyzing the impact of each one point increase in allostatic load prior and post to the cancer diagnosis in the risk of developing a 2-year major cardiac event (MACE) and its subtypes (heart failure, ischemic stroke, acute coronary syndrome, and atrial fibrillation) in non-hispanic Blacks (NHB) and non-hispanic Whites (NHW). Results are presented in adjusted hazard ratios (aHR), and 95% confidence intervals (CI). Multivariable models were adjusted for age at diagnosis, race, smoking status, surgery, radiotherapy, ADT, Elixhauser`s score, % of appointments attended and cardiovascular risk factors. All models, except those marked, reached p<0.05.

	**Prostate Cancer non-Hispanic Black (NHB) population (n=1,278)**							
	**Pre-cancer diagnostic allostatic load**				**Post-diagnosis allostatic load**			
	**Univariable**		**Multivariable**		**Univariable**		**Multivariable**	
	**aHR**	**95% CI**	**aHR**	**95% CI**	**aHR**	**95% CI**	**aHR**	**95% CI**
**MACE**	1.35	1.26-1.44	1.25	1.13-1.38	1.36	1.27-1.45	1.27	1.16-1.40
**Heart Failure**	1.37	1.24-1.51	1.35	1.14-1.60	1.36	1.23-1.51	1.35	1.13-1.51
**Ischemic Stroke**	1.17	1.05-1.29	1.13*	0.96-1.33	1.22	1.11-1.34	1.23	1.07-1.41
**Acute Coronary Syndrome**	1.41	1.27-1.57	1.33	1.10-1.61	1.42	1.28-1.57	1.31	1.09-1.57
**Atrial Fibrillation**	1.33	1.16-1.52	1.10**	0.89-1.36	1.34	1.18-1.52	1.12***	0.92-1.37
	**Prostate Cancer non-Hispanic White (NHW) UH population (n=3,478)**							
**MACE**	1.46	1.39-1.53	1.25	1.16-1.35	1.47	1.40-1.54	1.26	1.17-1.36
**Heart Failure**	1.47	1.38-1.58	1.17	1.03-1.07	1.47	1.38-1.58	1.2	1.06-1.35
**Ischemic Stroke**	1.54	1.40-1.71	1.29	1.07-1.55	1.55	1.40-1.71	1.31	1.10-1.57
**Acute Coronary Syndrome**	1.54	1.41-1.67	1.38	1.17-1.61	1.57	1.44-1.71	1.4	1.19-1.64
**Atrial Fibrillation**	1.34	1.24-1.45	1.2	1.07-1.35	1.36	1.26-1.47	1.2	1.07-1.34

*p=0.13

**p=0.34

***p=0.23

Supplementary Table 5. Fine and Gray competing risk regressions analyzing the impact of each one point increase in allostatic load prior and post to the cancer diagnosis in the risk of developing a 2-year major cardiac event (MACE) and its subtypes (heart failure, ischemic stroke, acute coronary syndrome and atrial fibrillation) in ADT, patients diagnosed > 2012, and allostatic load calculation via an alternative method. Results are presented in hazard ratios (HR), and 95% confidence intervals (CI). Multivariable models were adjusted for age at diagnosis, race, smoking status, surgery, radiotherapy, ADT, Elixhauser`s score, % of appointments attended and cardiovascular risk factors. All models, except those marked, reached p<0.05.

	**Prostate Cancer ADT population (n=1,178)**							
	**Pre-cancer diagnostic allostatic load**				**Post-diagnosis allostatic load**			
	**Univariable**		**Multivariable**		**Univariable**		**Multivariable**	
	**HR**	**95% CI**	**HR**	**95% CI**	**HR**	**95% CI**	**HR**	**95% CI**
**MACE**	1.35	1.26-1.45	1.26	1.16-1.38	1.35	1.26-1.45	1.27	1.17-1.39
**Heart Failure**	1.43	1.29-1.59	1.28	1.09-1.50	1.43	1.28-1.58	1.29	1.10-1.51
**Ischemic Stroke**	1.3	1.15-1.47	1.2	1.02-1.42	1.31	1.16-1.48	1.23	1.05-1.45
**Acute Coronary Syndrome**	1.45	1.29-1.63	1.29	1.09-1.53	1.45	1.29-1.63	1.31	1.11-1.55
**Atrial Fibrillation**	1.2	1.07-1.35	1.15*	0.99-1.34	1.2	1.07-1.35	1.17	1.02-1.36
	**Prostate Cancer diagnosis > 2012 (n=3,454)**							
**MACE**	1.28	1.22-1.34	1.1	1.02-1.19	1.29	1.23-1.35	1.11	1.03-1.20
**Heart Failure**	1.31	1.23-1.39	1.23	1.11-1.37	1.3	1.22-1.39	1.12	1.01-1.25
**Ischemic Stroke**	1.23	1.12-1.35	1.01***	0.87-1.17	1.26	1.15-1.37	1.17	1.03-1.33
**Acute Coronary Syndrome**	1.37	1.27-1.48	1.26	1.09-1.46	1.39	1.29-1.51	1.27	1.09-1.47
**Atrial Fibrillation**	1.19	1.10-1.28	1.13	1.01-1.26	1.2	1.10-1.29	1.12	1.01-1.26
	**Alostatic load by Chen et al. and Parente et al. method**							
**MACE**	1.63	1.53-1.73	1.34	1.22-1.48	1.67	1.57-1.77	1.4	1.27-1.54
**Heart Failure**	1.52	1.42-1.62	1.41	1.23-1.61	1.52	1.43-1.62	1.33	1.16-1.53
**Ischemic Stroke**	1.38	1.23-1.53	1.48**	0.98-1.36	1.43	1.30-1.57	1.31	1.13-1.50
**Acute Coronary Syndrome**	1.54	1.41-1.68	1.37	1.16-1.63	1.56	1.43-1.71	1.38	1.15-1.65
**Atrial Fibrillation**	1.38	1.28-1.48	1.28	1.15-1.43	1.41	1.31-1.51	1.3	1.17-1.45

***p=0.06**

****p=0.07**

*****p=0.85**

Supplementary Table 6. Subgroup and sensitivity analysis of multivariable piecewise Cox model with 4 follow-up time segments related to cancer diagnosis date (before, 2 months after, 6 months after and 1-year after) accounting for the effect of allostatic load variation in 2-year major cardiac event (MACE). Results are presented in adjusted hazard ratios (aHR), 95% confidence intervals (CI), and p-values. Multivariable models were adjusted for age at diagnosis, race, smoking status, surgery, radiotherapy, ADT, Elixhauser`s score, % of appointments attended and cardiovascular risk factors. HF=heart failure; IS=ischemic stroke; ACS=acute coronary syndrome; A-fib=atrial fibrillation. All models reached p<0.05.

	**Prostate Cancer non-Hispanic Black (NHB) population (n=1,278)**									
	**2-year MACE**		**2-year HF**		**2-year IS**		**2-year ACS**		**2-year A-fib**	
	**aHR**	**95% CI**	**aHR**	**95% CI**	**aHR**	**95% CI**	**aHR**	**95% CI**	**aHR**	**95% CI**
**AL Before diagnosis**	1.22	1.06-1.40	1.44	1.34-1.54	1.31	1.21-1.41	1.42	1.32-1.54	1.11	1.03-1.21
**AL 2 months after diagnosis**	1.26	1.10-1.45	1.44	1.34-1.54	1.32	1.22-1.43	1.43	1.31-1.55	1.12	1.03-1.21
**AL 2-6 months after diagnosis**	1.33	1.22-1.41	1.45	1.34-1.56	1.33	1.22-1.44	1.43	1.31-1.56	1.12	1.02-1.22
**AL 6-12 months after diagnosis**	1.34	1.26-1.42	1.44	1.31-1.58	1.35	1.20.150	1.44	1.29-1.61	1.11	1.01-1.25
**Prostate Cancer non-Hispanic White (NHW) population (n=3,478)**										
**AL Before diagnosis**	1.16	1.06-1.27	1.24	1.19-1.30	1.39	1.27-1.52	1.46	1.39-1.54	1.26	1.21-1.32
**AL 2 months after diagnosis**	1.2	1.10-1.31	1.25	1.19-1.31	1.4	1.27-1.53	1.47	1.39-1.55	1.27	1.22-1.32
**AL 2-6 months after diagnosis**	1.27	1.21-1.33	1.25	1.19-1.31	1.4	1.27-1.54	1.47	1.39-1.55	1.28	1.22-1.33
**AL 6-12 months after diagnosis**	1.31	1.26-1.36	1.26	1.18-1.34	1.39	1.28-1.51	1.46	1.36-1.57	1.29	1.22-1.37
**Prostate Cancer ADT population (n=1,178)**										
**AL Before diagnosis**	1.16	1.05-1.28	1.39	1.31-1.46	1.34	1.26-1.42	1.41	1.34-1.49	1.23	1.17-1.30
**AL 2 months after diagnosis**	1.2	1.08-1.32	1.39	1.31-1.47	1.35	1.27-1.43	1.42	1.34-1.51	1.24	1.17-1.31
**AL 2-6 months after diagnosis**	1.26	1.20-1.33	1.4	1.32-1.49	1.36	1.27-1.45	1.42	1.34-1.51	1.25	1.18-1.32
**AL 6-12 months after diagnosis**	1.31	1.25-1.36	1.41	1.31-1.53	1.37	1.26-1.49	1.43	1.33-1.54	1.27	1.18-1.37
**Prostate Cancer diagnosis > 2012 (n=3,454)**										
**AL Before diagnosis**	1.1	1.01-1.20	1.07	1.02-1.12	1.06	1.01-1.13	1.28	1.22-1.34	1.1	1.05-1.15
**AL 2 months after diagnosis**	1.15	1.05-1.26	1.08	1.03-1.13	1.07	1.01-1.14	1.28	1.22-1.35	1.01	1.01-1.02
**AL 2-6 months after diagnosis**	1.23	1.17-1.28	1.08	1.03-1.14	1.11	1.03-1.20	1.29	1.22-1.36	1.1	1.05-1.17
**AL 6-12 months after diagnosis**	1.26	1.22-1.31	1.07	1.01-1.15	1.12	1.01-1.25	1.28	1.19-1.38	1.11	1.30-1.19
**Alostatic load by Chen et al. and Parente et al. method**										
**AL Before diagnosis**	1.23	1.09-1.37	1.57	1.47-1.67	1.56	1.44-1.68	1.59	1.49-1.70	1.32	1.25-1.39
**AL 2 months after diagnosis**	1.3	1.16-1.46	1.6	1.49-1.70	1.57	1.45-1.70	1.6	1.49-1.71	1.32	1.24-1.39
**AL 2-6 months after diagnosis**	1.39	1.31-1.47	1.61	1.50-1.72	1.58	1.45-1.71	1.6	1.49-1.72	1.32	1.24-1.40
**AL 6-12 months after diagnosis**	1.44	1.37-1.51	1.62	1.48-1.77	1.61	1.44-1.79	1.6	1.46-1.76	1.32	1.22-1.42
